# Trapping and retaining intermediates in glycosyltransferases

**DOI:** 10.1016/j.jbc.2023.105006

**Published:** 2023-07-01

**Authors:** Marcelo E. Guerin

**Affiliations:** Department of Structural and Molecular Biology, Structural Glycobiology Laboratory, Molecular Biology Institute of Barcelona (IBMB), Spanish National Research Council (CSIC), Barcelona, Catalonia, Spain

**Keywords:** *Escherichia coli*, cell surface, capsular polysaccharide, glycolipid biosynthesis, glycosyltransferase, CAZyme, enzyme structure, enzyme mechanism, enzyme catalysis

## Abstract

Glycosyltransferases (GTs) attach sugar molecules to a broad range of acceptors, generating a remarkable amount of structural diversity in biological systems. GTs are classified as either “retaining” or “inverting” enzymes. Most retaining GTs typically use an S_N_i mechanism. In a recent article in the JBC, Doyle *et al.* demonstrate a covalent intermediate in the dual-module KpsC GT (GT107) supporting a double displacement mechanism.

After a brilliant series of chemical analyses, Luis F. Leloir and colleagues discovered UDP-glucose, for which he won the Nobel Prize in 1970. They soon isolated other members of the sugar nucleotide family and demonstrated that these compounds act as sugar donors in the biosynthesis of a broad range of natural disaccharides, oligosaccharides, polysaccharides, and glycoconjugates (1). We know now that organisms from all domains of life utilize sugar-nucleotide-dependent glycosyltransferases (GTs) for the synthesis of glycosidic bonds. Although sugar-nucleotide-dependent GTs use a restricted set of donor molecules, the adaptability of such enzymes to recognize a myriad of acceptor substrates results in substantial structural diversity of biosynthetic products, important not only for the maintenance of the cellular structure and biological processes but also in the chemoenzymatic synthesis of glycans and glycoengineering.

Monosaccharides exist in two anomeric forms, α- and β-anomers (2). The sugar nucleotide donor substrates of GTs as well as their reaction products exist in either one or the other anomeric forms. If as a result of the action of a GT, they are the same—for example, both the sugar nucleotide substrate and the resulting product are in the α-linked form—the GTs are classified as “retaining,” but if different, the GTs are called “inverting” (3, 4) (Fig. 1*A*). Most “inverting” sugar-nucleotide-dependent GTs utilize an S_N_2 single-displacement reaction mechanism in which the acceptor nucleophilic hydroxyl oxygen attacks the donor sugar anomeric carbon and displaces the leaving group nucleotide moiety from the opposite face, involving the formation of an oxocarbenium ion-like transition state (4). These enzymes employ a catalytic base, typically Glu, Asp, or His, which assist by deprotonating the nucleophile. For a few inverting sugar-nucleotide-dependent GTs without an identifiable catalytic base, an S_N_1 reaction mechanism has also been proposed (4).

**Figure 1:**
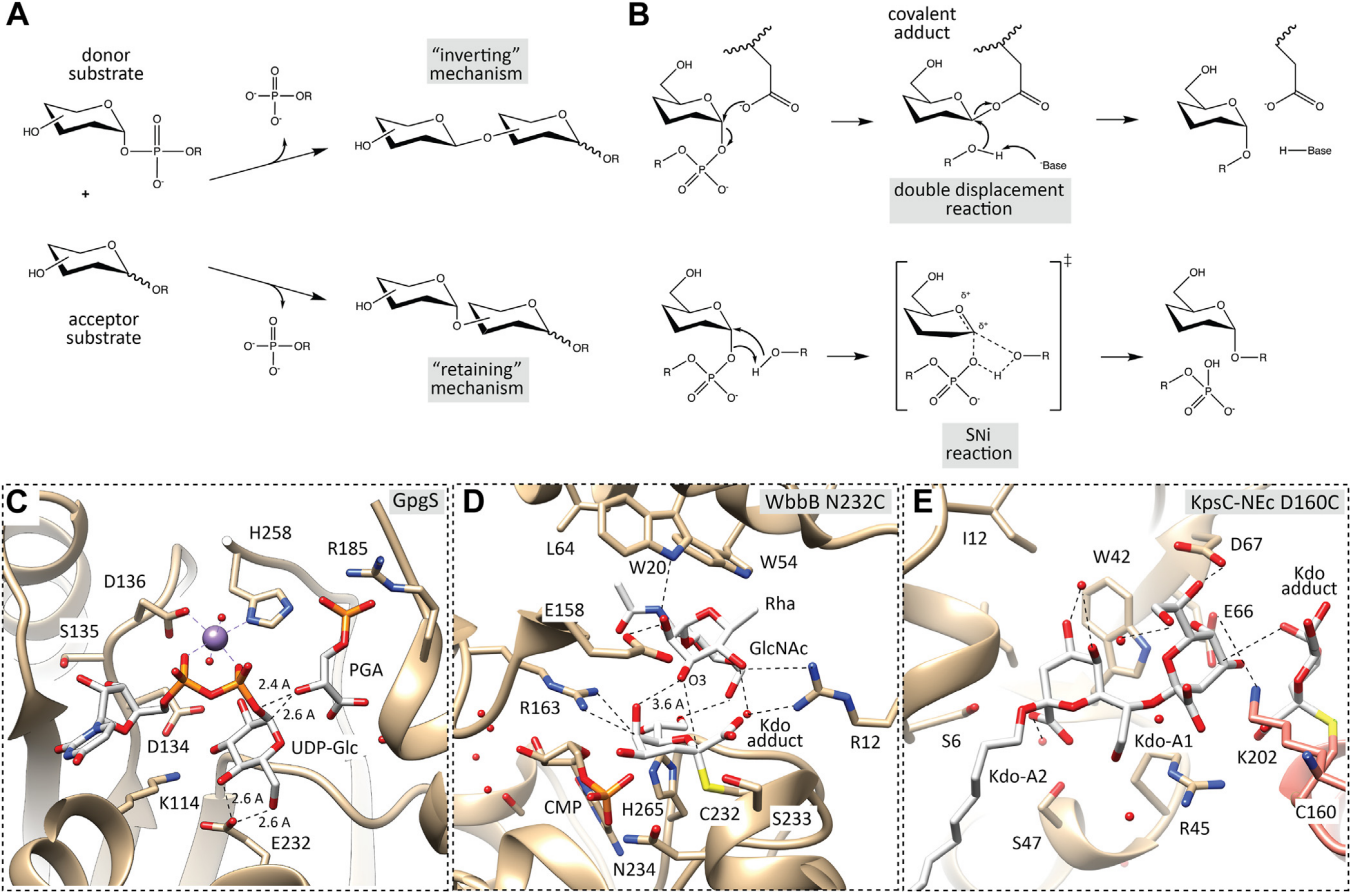
**Proposed reaction mechanisms for ‘retaining’ GTs.***A*, GT enzymes are classified in ‘retaining’ or ‘inverting’ depending of the anomeric configuration of reactants and products. *B*, two potential mechanisms have been proposed for ‘retaining’ GTs, a double-displacement and an S_N_i mechanisms. *C*, ternary complex of a ‘retaining’ GT enzyme following the proposed S_N_i mechanism: GpgS (PDB 4Y6N) (8). *D*, details of the interactions made by the WbbB Asn232Cys mutant with the α-Kdo covalent intermediate adduct and acceptor disaccharide in the active site (PDB 8CSF) (9). *E*, details of the interactions made by the KpsC-NEc Asp160Cys mutant in complex with CMP, the Kdo covalent intermediate adduct and the acceptor in the active site (PDB 8FUX) (10).

The reaction mechanism for “retaining” GTs, however, has been a matter of debate. By analogy with glycoside hydrolases, a double-displacement mechanism involving a covalently bound glycosyl-enzyme intermediate was initially proposed (2, 3, 4, 5) (Fig. 1*B*). This mechanism requires the existence of a correctly positioned nucleophile, typically Glu or Asp, performing an S_N_2 single-displacement reaction, forming a covalent glycosyl-enzyme intermediate during the first part of the reaction, resulting in a first anomeric inversion. In the second part of the reaction, the activated acceptor substrate performs a second S_N_2 single-displacement reaction at the glycosyl-enzyme intermediate, resulting in a second inversion and the formation of a product with total retention of the original anomeric configuration (2). To date, only members of GT6 family have been predicted to display such a putative nucleophile (3, 5). Quantum mechanics/molecular mechanics (QM/MM) metadynamics analysis of the GT mechanism of bovine GT6 α1,3-galactosyltransferase (α3GalT) predicts that the donor saccharide forms an intermediate Glu-Gal covalent adduct prior to transfer to the acceptor (5). However, experiments in human GTA/GTB GT6s found that mutating Glu303 to Cys or Asp only slightly slows down the reaction. Native ternary complexes of α3GalT in the presence of UDP-Gal, lactose, and the divalent cation cofactor revealed the substrates are organized very similarly to other “retaining” GTs, with the putative nucleophile Glu317 participating in a hydrogen bond with the acceptor β-Gal O4 atom, supporting a role in acceptor binding (6). It is worth noting that subtle alterations in the environment of the active site of the enzyme may influence which mechanism is observed (5). A covalent intermediate, a requirement for the double-displacement mechanism, has never been demonstrated in any retaining GTs, at least up until recently. In the absence of such a residue in most sugar-nucleotide-dependent GTs, an unusual single-displacement mechanism named as “front-face” or S_N_i, substitution nucleophilic internal-like mechanism, was also proposed (Fig. 1*B*). In this mechanism, the acceptor hydroxyl nucleophile is deprotonated by the donor β-phosphate oxygen and attacks the anomeric carbon atom of the sugar donor from the same side as the leaving nucleotide, and involves a short-lived oxocarbenium ion intermediate (4, 5). Several structural snapshots of enzyme-donor-acceptor complexes for ‘retaining’ GTs strongly support this S_N_i-type mechanism (4, 7, 8) (Fig. 1*C*).

Very recently, mass spectrometry analyses of WbbB from GT family 99, a retaining GT that adds a terminal β-Kdo (3-deoxy-D-manno-oct-2-ulosonic acid) residue to the O-antigen saccharide, identified a covalent adduct between the catalytic nucleophile, Asp232, and Kdo (9). Crystal structures show that the enzyme-linked Asp232-Kdo adduct rotates to reposition the Kdo into a second subsite, which then transfers the Kdo moiety to the acceptor (Fig. 1*D*). Doyle and colleagues identified the formation of covalent adducts with Kdo in a member of the GT107 family, KpsC, a dual-module retaining GT that is essential for the biosynthesis of ‘group 2’ capsular polysaccharides in *Escherichia coli* and other Gram-negative pathogens (10). Crystal structures of a KpsC-NEc Asp160Asn mutant in complex with the α-Kdo adduct and of a second KpsC-NEc Asp160Cys mutant in complex with CMP, the Kdo adduct, and the acceptor support a double displacement mechanism rather than the generally observed S_N_i mechanism (Fig. 1*E*). Furthermore, they also show the adduct undergoes significant structural re-arrangement after transfer of the Kdo moiety from CMP. Comparison of the Kdo adduct of KpsC-NEc Asp160Asn with the equivalent WbbB Asp232Asn mutant (9, 10) reveals very similar positioning and orientation of the Kdo adduct in each active site, although none of the coordinating groups between the two enzymes is conserved. This suggests that despite the considerable divergence between KpsC (GT107) and WbbB (GT99), this conserved presentation mode of Kdo in the active site is likely a key feature of the mechanism in both enzyme families. The evolutionary origins of these enzymes as well as the development of specific inhibitors remain fascinating areas of research that encourages further investigation and exploration (9, 10).

## Conflict of interest

The authors declare that they have no conflicts of interest with the contents of this article.
